# Current spectral norm and phase variation based fault region identification for active distribution network

**DOI:** 10.1038/s41598-024-62859-6

**Published:** 2024-06-02

**Authors:** Jie Chen, Yong Li, Rong Zeng, Junle Liu, An Chen, Liang Hou, Long Zhao, Mohammad Shahidehpour

**Affiliations:** 1 College of Electrical and Information Engineering, Hunan University, Changsha, 410082 China; 2 China Southern Power Grid Guangdong Zhongshan Power Supply Bureau, Zhongshan, 528400 China; 3 CYG Sunri Co., Ltd., Shenzhen, 518000 China; 4 Electrical and Computer Engineering, Illinois Institute of Technology, Chicago, IL 60616-3793 USA

**Keywords:** Engineering, Electrical and electronic engineering

## Abstract

The paper presents a fault region identification method for the active distribution network (ADN) with limited PMU. First, PMU configuration and region division strategies are proposed based on the network topology. Next, the difference in three-phase current variations between the normal and fault regions of the ADN is analyzed. A multi-dimensional state monitoring matrix is built using the measured current data. The spectral norm ratio coefficient is constructed based on the 2-norm to lower the complexity of the multi-dimensional state monitoring matrix and quantify the difference in state changes before and after the fault occurs in each region. Then, the fault region is identified by combining each region’s spectral norm ratio coefficient and the change of the current phase. Finally, an IEEE 33-node simulation model is created to simulate faults under different working conditions. According to the simulation results, the suggested approach is less impacted by fault type, neutral point grounding mode, and transition resistance. Furthermore, even if the communication does not match the rigorous synchronization requirements, the proposed method can still complete the fault identification of the distribution network correctly and has high robustness.

## Introduction

Active distribution network (ADN) is directly connected with users, which is the key link to ensure the quality of power supply and user service and improve the operation efficiency of power system. With the access of a large number of distributed energy resources such as photovoltaic solar, wind, and battery storage, the complexity of traditional fault location technology of single power system in active distribution network is also increasing. Therefore, it is urgent to propose an effective and fast fault location method for active distribution network.

The fault location methods of active distribution network are mainly based on electrical quantity, impedance, traveling wave method and intelligent algorithm. The method based on electrical quantities usually uses the positive sequence^1,2^ or zero sequence^3–5^ quantities during the fault to estimate the fault distance. However, too high transition resistance will make the change of electrical quantity not obvious, which will lead to the failure of location method. The impedance-based method mainly locates the fault by constructing the impedance matrix^6–8^. But the main flaw in the suggested techniques is that the impedance matrix construction cannot be done offline since the network structure changes due to sub-network formation and network reconfiguration. Thus, creating an impedance matrix does not significantly benefit large systems but increases complexity. The traveling wave method mainly uses the arrival time of the incident wave and the reflected wave to estimate the fault distance^9,10^. However, it must maintain the timing of measurements. What’s more, it requires high sampling frequencies and can be adversely affected by noise-corrupted measurements. The intelligent algorithm mainly uses the related optimization algorithm to realize the fault location. Refs.^11–13^ use Bayesian compressed sensing (BCS) to pinpoint all fault types in distribution networks. However, this method makes it easy to locate the adjacent regions of the fault region.

Phasor Measurement Units (PMUs) are being used increasingly often due to the ongoing advancements in communication technology, and they have grown to be a crucial component of the dynamic power system monitoring process. It obtains voltage and current phase data mainly by the discrete Fourier variation method. Based on the measurement data obtained by the PMU, it offers significant data support for the location of distribution network faults and effectively satisfies the demands for quick and precise fault location. Using the voltage threshold value determined by a thorough investigation of the test system, Reference^14^ uses the data from the optimal PMU locations to provide a two-stage fault detection method. However, does not specifically address how to get the optimal PMU location. Reference^15^ investigates a new field of study for defective region detection with little data from sensors in the power distribution system to address the issue of a large variety of PMU setups. In addition, literatures^16–18^ classify and locate the defects using deep learning algorithms based on measured data. The solutions, however, will not operate in a distribution network system with DGs and are only helpful for traditional distribution networks.

This paper analyzed the fault characteristics in ADN. Based on the characteristics of current variation before and after the fault occurs, the spectral norm ratio coefficient (SNRC) and current phase difference (CPD) are defined. The proposed method only needs current amplitude and phase data of any period before and after the fault occurs, so it can avoid the impact of time synchronization on fault region identification. Moreover, the method is not affected by fault type, neutral point grounding type, and transition resistance.

The remaining of this article is as follows: “Fault features of active distribution network” section analyzes current features in ADN. How to implement fault region identification using the SNRC and CPD is described in “Fault region identification method” section. “Simulation results” section uses PSCAD and MATLAB to verify the effectiveness of the proposed method from different fault position, fault types, neutral point grounding methods, and transition resistances. “Experiment results” section verifies the practical significance of the method. The conclusion is presented in “Conclusion” section.

## Fault features of active distribution network

### Fault features of distribution network without DG

**Figure 1:**
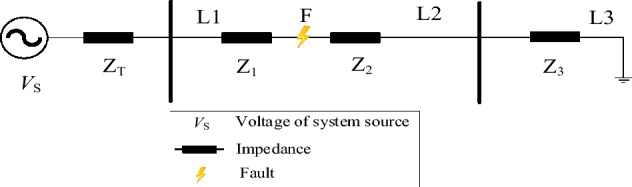
Distribution network without DG.

Figure 1 illustrates this by using the A-phase ground fault at fault point F as an example (for research, L1 and L2 are set as the upstream and downstream lines of point F, respectively). The present relationship between lines L1, L2, and L3 is satisfied after the fault occurs as follows:

1 $$\left\{ \begin{aligned} & I_{{1{\text{AF}}}} {\kern 1pt} = I_{{f{\text{A}}}} + V_{{f{\text{A}}}} /Z_{{\text{d}}} \hfill \\ & I_{{{\text{1BF}}}} {\kern 1pt} = I_{{f{\text{B}}}} + V_{{f{\text{B}}}} /Z_{{\text{d}}} \hfill \\ & I_{{1C{\text{F}}}} = V_{{f{\text{C}}}} /Z_{{\text{d}}} \hfill \\ \end{aligned} \right.$$

where ***I***_1AF_, ***I***_1BF,_ and ***I***_1CF_ are the A, B, and C three-phase currents of line L1, respectively. ***I***_*f*A_, ***I***_*f*B,_ and ***I***_*f*C_ are the fault currents of the A, B, and C phases at the fault point F, respectively. ***V***_*f*A_, ***V***_*f*B,_ and ***V***_*f*C_ are the fault voltages of A, B, and C phases at the fault point F, respectively. *Z*_d_ is the sum of *Z*_2_ and *Z*_3_.

When an A-phase grounding fault occurs, there are:

2 $$\left\{ \begin{aligned} & I_{{f{\text{A}}}} = \frac{{3V_{f}^{(0)} }}{{Z^{(1)} + Z^{(2)} + Z^{(0)} }} \hfill \\ & I_{{f{\text{B}}}} = I_{{f{\text{C}}}} = 0 \hfill \\ & V_{{f{\text{B}}}} = \frac{{(a^{2} - a)Z^{(2)} + (a^{2} - 1)Z^{(0)} }}{{Z^{(1)} + Z^{(2)} + Z^{(0)} }}V_{f}^{(0)} \hfill \\ & V_{{f{\text{C}}}} = \frac{{(a - a^{2} )Z^{(2)} + (a - 1)Z^{(0)} }}{{Z^{(1)} + Z^{(2)} + Z^{(0)} }}V_{f}^{(0)} \hfill \\ & V_{{f{\text{A}}}} = 0 \hfill \\ \end{aligned} \right.$$

where ***V***_*f*_^(0)^ is the voltage before fault at fault point F and *a* is equal to *e*^j120^. *Z*^(1)^ is the sum of equivalent positive sequence impedance in line, *Z*^(2)^ is the sum of equal negative sequence impedance in line, and *Z*^(0)^ is the sum of equivalent zero sequence impedance in line. In this paper, *Z*^(1)^ equals *Z*^(2)^.

Combined with Eqs. (1)-(2), the currents of L1_A_, L1_B_, and L1_C_ after fault are:

3 $$\left\{ \begin{aligned} & I_{{1{\text{AF}}}} {\kern 1pt} = \frac{{3V_{f}^{(0)} }}{{Z^{(1)} + Z^{(2)} + Z^{(0)} }} \hfill \\ & I_{{1{\text{BF}}}} {\kern 1pt} = \frac{{(a^{2} - a)Z^{(2)} + (a^{2} - 1)Z^{(0)} }}{{Z_{{\text{d}}} (Z^{(1)} + Z^{(2)} + Z^{(0)} )}}V_{f}^{(0)} \hfill \\ & I_{{1{\text{CF}}}} {\kern 1pt} = \frac{{(a - a^{2} )Z^{(2)} + (a - 1)Z^{(0)} }}{{Z_{{\text{d}}} (Z^{(1)} + Z^{(2)} + Z^{(0)} )}}V_{f}^{(0)} \hfill \\ \end{aligned} \right.$$

The currents of line L2 and L3 after the A-phase fault are:

4 $$\left\{ \begin{aligned} I_{{2{\text{BF}}}} {\kern 1pt} = I_{{{\text{3BF}}}} = \frac{{(a^{2} - a)Z^{(2)} + (a^{2} - 1)Z^{(0)} }}{{Z_{{\text{d}}} (Z^{(1)} + Z^{(2)} + Z^{(0)} )}}V_{f}^{(0)} \hfill \\ I_{{2{\text{CF}}}} {\kern 1pt} = I_{{{\text{3CF}}}} = \frac{{(a - a^{2} )Z^{(2)} + (a - 1)Z^{(0)} }}{{Z_{{\text{d}}} (Z^{(1)} + Z^{(2)} + Z^{(0)} )}}V_{f}^{(0)} \hfill \\ \end{aligned} \right.$$

Thus, the current variation of L1_B_, L1_C_, L2, and L3 are:

5 $$\left\{ \begin{aligned} & \Delta I_{1B} {\kern 1pt} = \Delta I_{2B} = \Delta I_{2B} = \frac{{(a^{2} - a)Z^{(2)} + (a^{2} - 1)Z^{(0)} }}{{Z_{{\text{d}}} (Z^{(1)} + Z^{(2)} + Z^{(0)} )}}V_{f}^{(0)} - \frac{{V_{{f{\text{B}}}}^{(0)} }}{{Z_{{\text{d}}} }} \hfill \\ & \Delta I_{1C} {\kern 1pt} = \Delta I_{2C} = \Delta I_{2C} = \frac{{(a - a^{2} )Z^{(2)} + (a - 1)Z^{(0)} }}{{Z_{{\text{d}}} (Z^{(1)} + Z^{(2)} + Z^{(0)} )}}V_{f}^{(0)} - \frac{{V_{{f{\text{C}}}}^{(0)} }}{{Z_{{\text{d}}} }} \hfill \\ & \Delta I_{{2{\text{A}}}} {\kern 1pt} = \Delta I_{{3{\text{A}}}} = - \frac{{V_{f}^{(0)} }}{{Z_{{\text{d}}} }} \hfill \\ \end{aligned} \right.$$

In the formula, ***V***_*f*B_^(0)^ and ***V***_*f*C_^(0)^ are the voltages of the B and C phases before fault at fault point F.

The current variation of lines L1_A_ is:

6 $$\Delta I_{{1{\text{A}}}} = \frac{{3V_{f}^{(0)} }}{{Z^{(1)} + Z^{(2)} + Z^{(0)} }} - \frac{{V_{f}^{(0)} }}{{Z_{{\text{d}}} }}$$

According to Eqs. (5), (6), the sum of the fault current and the phase current downstream of the fault site is the fault phase current upstream of the fault point.

When a B and C two-phase ground short circuit occurs at point F, the current of line L1 may be determined as follows:

7 $$\left\{ \begin{aligned} & I_{{{\text{1BF}}}} {\kern 1pt} = I_{{f{\text{B}}}} + \frac{{V_{{f{\text{B}}}} }}{{Z_{{\text{d}}} }} \hfill \\ & I_{{{\text{1CF}}}} {\kern 1pt} = I_{{f{\text{C}}}} + \frac{{V_{{f{\text{C}}}} }}{{Z_{{\text{d}}} }} \hfill \\ & I_{{{\text{1AF}}}} = \frac{{V_{{f{\text{A}}}} }}{{Z_{{\text{d}}} }} \hfill \\ \end{aligned} \right.$$

where, ***I***_1AF_, ***I***_1BF,_ and ***I***_1CF_ are the A, B, and C three-phase currents of line L1, respectively. ***I***_*f*B_ and ***I***_*f*C_ are the fault currents of the B and C phase at the fault point F, respectively. ***V***_*f*A_, ***V***_*f*B,_ and ***V***_*f*C_ are the fault voltages of A, B, and C phases at the fault point F, respectively. *Z*_d_ is the equivalent impedance from fault point F to the end of the line.

When a BC two-phase ground short circuit occurs in the distribution network, there are:

8 $$\left\{ \begin{aligned} & I_{{f{\text{B}}}} = (a^{2} - \frac{{Z^{(2)} + aZ^{(0)} }}{{Z^{(2)} + Z^{(0)} }})I_{{f{\text{A(1)}}}} \hfill \\ & I_{{f{\text{C}}}} = (a - \frac{{Z^{(2)} + a^{2} Z^{(0)} }}{{Z^{(2)} + Z^{(0)} }})I_{{f{\text{A(1)}}}} \hfill \\ & V_{{f{\text{B}}}} = V_{{f{\text{C}}}} = 0 \hfill \\ & I_{{f{\text{A(1)}}}} = \frac{{V_{f}^{(0)} }}{{Z^{(1)} + Z_{{\text{p}}} }} \hfill \\ & V_{{f{\text{A}}}} = \frac{{3Z^{(2)} Z^{(0)} }}{{Z^{(2)} + Z^{(0)} }}I_{{f{\text{A(1)}}}} \hfill \\ \end{aligned} \right.$$

where ***I***_*f*B_ and ***I***_*f*C_ are the fault currents of B and C phases at fault point F. ***V***_*f*A_, ***V***_*f*B_, and ***V***_C_ are the fault voltages of the A, B, and C phases at fault point F. ***I***_*f*A(1)_ is the positive sequence current of the A phase in line L1 and *a* is equal to *e*^j120^, ***V***_*f*_^(0)^ is the voltage before fault at fault point F. *Z*^(1)^ is the sum of equivalent positive sequence impedance in line, *Z*^(2)^ is the sum of equal negative sequence impedance in line, and *Z*^(0)^ is the sum of equivalent zero sequence impedance in line. *Z*_P_ is the parallel equivalent resistance of *Z*^(2)^ and *Z*^(0)^. In this paper, *Z*^(1)^ equals *Z*^(2)^.

Combined with Eqs. (7)-(8), the currents of L1_A_, L1_B_, and L1_C_ after fault are:

9 $$\left\{ \begin{aligned} & I_{{{\text{1BF}}}} {\kern 1pt} = V_{f}^{(0)} \frac{{(a^{2} - 1)Z^{(1)} + (a^{2} - a)Z^{(0)} }}{{(Z^{(1)} )^{2} + 2Z^{(1)} Z^{(0)} }} \hfill \\ & I_{{{\text{1CF}}}} {\kern 1pt} = V_{f}^{(0)} \frac{{(a - 1)Z^{(1)} + (a - a^{2} )Z^{(0)} }}{{(Z^{(1)} )^{2} + 2Z^{(1)} Z^{(0)} }} \hfill \\ & I_{{{\text{1AF}}}} = V_{f}^{(0)} \frac{{3Z^{(1)} Z^{(0)} }}{{Z_{d} ((Z^{(1)} )^{2} + 2Z^{(1)} Z^{(0)} )}} \hfill \\ \end{aligned} \right.$$

The currents of line L2 and L3 after the B, and C two-phase ground fault are:

10 $$\left\{ \begin{aligned} & I_{{{\text{2BF}}}} {\kern 1pt} = I_{{{\text{2CF}}}} = I_{{3{\text{BF}}}} {\kern 1pt} = I_{{3{\text{CF}}}} = 0 \hfill \\& I_{{{\text{2AF}}}} = I_{{3{\text{AF}}}} = V_{f}^{(0)} \frac{{3Z^{(1)} Z^{(0)} }}{{Z_{{\text{d}}} ((Z^{(1)} )^{2} + 2Z^{(1)} Z^{(0)} )}} \hfill \\ \end{aligned} \right.$$

Therefore, the current variation of L1_A_, L2, and L3 is:

11 $$\left\{ \begin{aligned} & \Delta I_{{{\text{1A}}}} {\kern 1pt} = \Delta I_{{{\text{2A}}}} = \Delta I_{{3{\text{A}}}} = V_{f}^{(0)} \frac{{Z^{(1)} Z^{(0)} - (Z^{(1)} )^{2} }}{{Z_{{\text{d}}} ((Z^{(1)} )^{2} + 2Z^{(1)} Z^{(0)} )}} \hfill \\ & \Delta I_{{2{\text{B}}}} {\kern 1pt} = \Delta I_{{{\text{3B}}}} {\kern 1pt} = - \frac{{V_{{f{\text{B}}}}^{(0)} }}{{Z_{{\text{d}}} }} \hfill \\ & \Delta I_{{2{\text{C}}}} {\kern 1pt} = \Delta I_{{{\text{3C}}}} {\kern 1pt} = - \frac{{V_{{f{\text{C}}}}^{(0)} }}{{Z_{{\text{d}}} }} \hfill \\ \end{aligned} \right.$$

where, ***V***_*f*B_^(0)^ and ***V***_*f*C_^(0)^ are the voltages of the B and C phases before fault at fault point F.

The current variation of lines L1_B_ and L1_C_ is:

12 $$\left\{ \begin{aligned} & \Delta I_{{2{\text{B}}}} {\kern 1pt} {\kern 1pt} = V_{f}^{(0)} \frac{{(a^{2} - 1)Z^{(1)} + (a^{2} - a)Z^{(0)} }}{{(Z^{(1)} )^{2} + 2Z^{(1)} Z^{(0)} }} - \frac{{V_{{f{\text{B}}}}^{(0)} }}{{Z_{{\text{d}}} }} \hfill \\ & \Delta I_{{2{\text{C}}}} {\kern 1pt} {\kern 1pt} = V_{f}^{(0)} \frac{{(a - 1)Z^{(1)} + (a - a^{2} )Z^{(0)} }}{{(Z^{(1)} )^{2} + 2Z^{(1)} Z^{(0)} }} - \frac{{V_{{f{\text{C}}}}^{(0)} }}{{Z_{{\text{d}}} }} \hfill \\ \end{aligned} \right.$$

Equations (10)-(12) demonstrate that when a two-phase fault occurs, the current of the fault phase downstream of the fault point is zero. The sum of the fault current and the phase current downstream of the fault point is the fault phase current upstream of the fault point. Because the voltage at the fault point is zero when a three-phase short-circuit fault occurs, the current downstream of the fault point is also zero. The current of the fault phase downstream of the fault point decreases when a phase-to-phase short-circuit fault occurs, and the mechanism is identical to a two-phase short-circuit fault. This paper will not be duplicated due to space limitations. In summary, when a fault occurs, the current of the fault phase upstream of the fault point is greater than the current of the fault phase downstream of the fault point.

### Fault features of distribution network with DG

**Figure 2:**
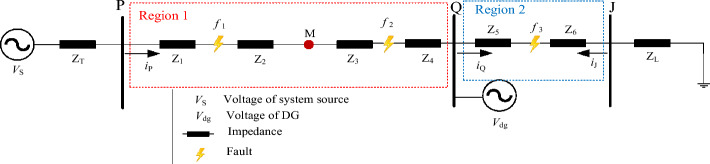
Distribution network with DG.

For the fault features of the downstream region without DG (i.e., the Region 2 in the Fig. 2), as shown in Fig. 2, when a fault occurs, the system power supply and DG will both provide a short-circuit current to the fault point.

Assuming that the positive direction is the direction of current flow out of the bus. At this time, the current variations of nodes Q and J before and after faults are:

13 $$\Delta i_{Q} = \Delta i_{s} + \Delta i_{dg}$$

14 $$\Delta i_{J} = - i_{J}$$

where, Δ*i*_s_ is the current variation the system power supply provides before and after the fault. Δ*i*_dg_ is the variation provided by DG, and *i*_J_ is the current at point J before the fault.

When a fault occurs in the Region 2, i.e., fault *f*_3_, the current variation downstream of the fault point equals the current before the fault, and the current variation upstream of the fault point equals the sum of the system power supply current variation and the current variation supplied by the DG. So, by contrasting the current amplitude difference before and after the fault, the fault region may be identified.

For the fault features of the downstream region with DG (i.e., the Region 1 in the Fig. 2), as shown in Fig. 2, when a fault occurs, the fault current from DG will flow to the fault point if DG is connected to the fault region. Point M of the Region 1 in Fig. 2 is arbitrarily chosen to study the current attributes of the upstream and downstream fault. The direction of the current flowing out of the bus is the positive direction.

When no fault occurs, the current ***I***_Mpre_ of the point M is:

15 $$I_{{{\text{Mpre}}}} = \frac{{V_{{\text{S}}} - V_{{\text{M}}} }}{{Z_{{\text{T}}} + Z_{1} + Z_{2} }}$$

***V***_S_ and ***V***_M_ are the system source and M-point voltage during the regular line operation. *Z*_T_ is the sum of the internal impedance of the transformer and the system source. *Z*_1_ and *Z*_2_ are the line impedances.

**Figure 3:**
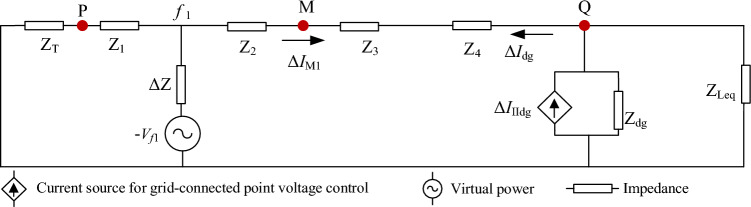
Equivalent circuit at *f*_1_ fault.
  The positive sequence current variation Δ***I***_M1_ of M point is:
  19 $$\Delta I_{{{\text{M1}}}} = - \Delta I_{dg}$$
  where, Δ***I***_dg_ is the variation of the output current of DG. When the inverter-type DG output current is equal to 1.2 p.u., the amplitude range of the positive sequence fault component before and after the M-point fault is obtained as follows:
  20 $$|\Delta I_{{{\text{dg}}}} | \in 0.22p.u.\sim 0.61p.u.$$
  Δ***I***_M1_ is negative, so the current of M point phase lags behind ***I***_Mpre_ phase after a fault. Assume that a BC two-phase short-circuit fault occurs downstream of point M, as depicted in *f*_2_ of Fig. 2. The equivalent circuit at *f*_2_ fault is shown in Fig. 4.

1. Single-phase fault feature in Region 1 of Fig. 2.
  Suppose that an A-phase fault occurs upstream of the detection point M, as shown in *f*_1_ of Fig. 2. The current ***I***_MF1_ of the point M is:
  16 $$I_{{{\text{MF1}}}} = \frac{{V_{f1} - V_{Q} }}{{Z_{{2}} + Z_{3} + Z_{4} }}$$
  where ***V***_*f*1_ and ***V***_Q_ are the fault point’s and Q point’s voltage, respectively. *Z*_2_, *Z*_3_, and *Z*_4_ are the line impedances.
  If the transition resistance is 0, it is obvious to find:
  17 $$\left\{ \begin{aligned} & V_{f1} = 0 \hfill \\ & I_{{{\text{MF1}}}} = \frac{{ - V_{Q} }}{{Z_{{2}} + Z_{3} + Z_{4} }} \hfill \\ \end{aligned} \right.$$
  Hence, ***I***_MF1_ is negative. the length of the line is generally not more than a few kilometers when the distribution network is operating normally, and the length of the line less impacts the impedance angle. It can be considered that the line impedance angle is roughly equal, ranging from 70° to 80°^19^. As a result, in Eq. (15), the amplitude of ***V***_S_-***V***_M_ is lower, and the phase is earlier than ***V***_S_. Transformer impedance and system impedance are both contained in *Z*_T_. Its maximum impedance angle is not more than 90°, so the phase of ***I***_MF1_ lies behind the phase of ***I***_Mpre_. If there is a transition resistance since the amplitude of the DG access point voltage is greater than ***V***_*f*1_, ***V***_*f*1_-***V***_Q_ is negative, and the phase of ***I***_MF1_ still lags behind the phase of ***I***_Mpre_.
  Assume that an A-phase fault develops downstream of point M, as shown in *f*_2_ of Fig. 2. The current ***I***_MF2_ of the point M is:
  18 $$I_{{{\text{MF2}}}} = \frac{{V_{{\text{S}}} - V_{f2} }}{{Z_{{\text{T}}} + Z_{1} + Z_{2} + Z{}_{3}}}$$
  where ***V***_*f*2_ and ***V***_S_ are the fault point and system source voltage, respectively. *Z*_1_, *Z*_2_, and *Z*_3_ are the line impedances. *Z*_T_ is the sum of the internal impedance of the system source and the impedance of the transformer.
  According to the study above, the phase of ***I***_MF2_ is ahead of the phase of ***I***_Mpre_ if the transition resistance is 0. On the other hand, the phase of ***V***_*f*2_ is behind the phase of the *f*_2_-point voltage when the line is operating normally. As a result, the phase of ***V***_*f*2_ is later than the phases of ***V***_M_ and ***V***_S_. It can be determined that the phase of ***I***_MF2_ is ahead of the phase of ***I***_Mpre_.
2. Two-phase short fault and three-phase short fault feature in Region 1 of Fig. 2.
  When a two-phase short-circuit fault occurs, the voltage phase of the access point will mutate. As a result, it is appropriate to use positive sequence quantity for analysis. Suppose that a BC two-phase short-circuit fault occurs upstream of point M, as illustrated in *f*_1_ of Fig. 2, and that the phase of the A-phase voltage at fault time is 0 degree as a reference. Figure 3 depicts the equivalent circuit at *f*_1_ fault.

**Figure 4:**
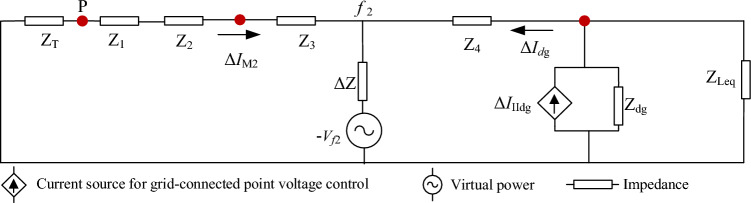
Equivalent circuit at *f*_2_ fault.
  According to the superposition theorem, the current positive sequence variation Δ***I***_M2_ of M can be obtained as:
  21 $$\begin{aligned} \Delta I_{{{\text{M2}}}} & = \frac{{V_{f2} }}{{\Delta Z + (Z_{T} + Z_{1} + Z_{2} + Z_{3} )||(Z_{4} + (Z_{{{\text{Leq}}}} ||Z_{dg} )}} \cdot \frac{{(Z_{4} + (Z_{{{\text{Leq}}}} ||Z_{dg} )}}{{Z_{T} + Z_{1} + Z_{2} + Z_{3} + Z_{4} + Z_{{{\text{Leq}}}} + Z_{dg} }} \hfill \\ & \quad - \Delta I_{dg} \cdot \frac{\Delta Z}{{\Delta Z + (Z_{T} + Z_{1} + Z_{2} + Z_{3} )}} \hfill \\ \end{aligned}$$
  where *Z*_dg_ is the internal impedance of DG and *Z*_Leq_ is the sum of the impedance downstream of the Region 1 and the load. Δ*Z* is the transition resistance. “||” is the parallel operation of impedance. For the virtual power branch, because the internal impedance of the inverter-type DG is infinite, it can be simplified as follows:
  22 $$\begin{aligned} \Delta I_{{{\text{M2}}}} & = \frac{{V_{f2} }}{{\Delta Z + (Z_{T} + Z_{1} + Z_{2} + Z_{3} )}} - \Delta I_{dg} \cdot \frac{\Delta Z}{{\Delta Z + (Z_{T} + Z_{1} + Z_{2} + Z_{3} )}} \hfill \\ & = \frac{{V_{f} - \Delta I_{dg} \Delta Z}}{{\Delta Z + (Z_{T} + Z_{1} + Z_{2} + Z_{3} )}} \hfill \\ \end{aligned}$$
  When a fault occurs, the amplitude of the positive-sequence voltage at the fault point is half what it was before the fault, and the voltage phase stays unaltered. The amplitude of the positive-sequence fault component of the virtual power supply's current is 3 to 12 times that of the DG's current. When the inverter-like DG’s output current is set at 1.2 p.u., according to reference^19^, there is:
  23 $$\left\{ \begin{aligned} & |\Delta I_{{{\text{M2}}}} | \in 2.86p.u.\sim 11.94p.u. \hfill \\ & |\Delta I_{{{\text{dg}}}} | \in 0.22p.u.\sim 0.61p.u. \hfill \\ \end{aligned} \right.$$
  Therefore, the direction of Δ***I***_M2_ is positive, implying that the current phase of M point is ahead of the phase of ***I***_Mpre_ after the fault. Taking the impedance angle of the overhead line as 72°^19^, the phase angle variation of the positive sequence fault component at both ends of the Region 1 may be calculated roughly as:
  24 $$\arg (\Delta I_{PQ} ) \in (0^{ \circ } ,87.3^{ \circ } )$$
  If a three-phase short-circuit fault occurs upstream and downstream of point M, it is seemingly possible to find:
  25 $$\left\{ \begin{aligned} & |\Delta I_{{{\text{M1}}}} | = |\Delta I_{{{\text{dg}}}} | \in 0.22pu\sim 2.0pu \hfill \\ & |\Delta I_{{{\text{M2}}}} | \in 5.58pu\sim 12.44pu \hfill \\ \end{aligned} \right.$$
  where |Δ***I***_M1_| is M point upstream fault current amplitude variation. |Δ***I***_M2_| is M point downstream fault current amplitude variation.
  Combining Eqs. (19), (22), and (25), it can be obtained that the phase of Δ***I***_M1_ is behind the phase of ***I***_Mpre_, whereas the phase of Δ***I***_M2_ is ahead of the phase of ***I***_Mpre_. Therefore, the phase angle variation of the positive sequence fault component at both ends of the Region 1 can be approximated as follows:
  26 $$\arg (\Delta I_{PQ} ) \in (0^{ \circ } ,162.9^{ \circ } )$$
  From above, when a fault occurs in a downstream region with DG access, the positive sequence fault current phase upstream of the fault point will be ahead of the normal operation phase. In contrast, the positive sequence fault current phase downstream of the fault point will lag. If we define *φ*_p_ as the phase before the fault and *φ*_F_ as the phase after the fault, then the phase variation ∆*φ* is:
  27 $$\Delta \varphi = \varphi_{{\text{p}}} - \varphi_{{\text{F}}}$$
  If ∆*φ* is positive, the phase after the fault is ahead of the phase before the fault. If ∆*φ* is negative, the phase after the fault lags behind the phase before the fault.
  When a fault occurs in the Region 2 in Fig. 2, there is ***I***_PF_ = ***I***_QF_. At both ends of the Region 1, the CPD of the positive fault sequence current is:
  28 $$\Delta \varphi_{PQ} = |\Delta \varphi_{P} - \Delta \varphi_{Q} | = 0$$
  The above analysis shows that the fault positive sequence current phase at upstream of the fault point is ahead of the phase before the fault. In contrast, the fault positive sequence current phase at downstream of the fault point lags behind the phase before the fault, resulting in the maximum amount of phase difference in faulty region. Equations (24) and (26) illustrate the range.

### Fault region identification method

#### Spectral norm ratio coefficient

The matrix 2-norm of the factual matrix **A**_*m*×*n*_ is defined as follows:

29 $$ \left\| {\bf {A}} \right\|_{2} = \sqrt {\max ({eig}({\bf {A}}^{{\text{T}}} \times {\bf {A}}))}$$

where **A** is an *m* × *n* matrix, **A**^**T**^ is a transposed matrix of **A**. *eig*(.) is the eigenvalue calculation function of a square matrix. *eig* (**A**^**T**^ × **A**) represents the eigenvector [λ_1_, …, λ_*i*_, …]^T^ of matrix **A**. λ_*i*_ represents the *i*th eigenvalue.

In this research, the length of the data acquisition time window is set to 10 ms. A fault is considered to have occurred when the current data acquired by the PMU exceeds the threshold. Data from each cycle before the fault is collected and discretized. The phase current matrix of node *i* before the fault is:

30 $$\left\{ \begin{aligned} & I_{{i\text{A}}} = [\begin{array}{*{20}c} {I_{{i\text{A}}} (1)} & {I_{{i\text{A}}} (2)} & {\begin{array}{*{20}c} \cdots & {I_{{i\text{A}}} (M)} \\ \end{array} } \\ \end{array} ]^{{\text{T}}} \\ & I_{{i{\text{B}}}} = [\begin{array}{*{20}c} {I_{{i{\text{B}}}} (1)} & {I_{{i{\text{B}}}} (2)} & {\begin{array}{*{20}c} \cdots & {I_{{i{\text{B}}}} (M)} \\ \end{array} } \\ \end{array} ]^{{\text{T}}} \\ & I_{{i{\text{C}}}} = [\begin{array}{*{20}c} {I_{{i{\text{C}}}} (1)} & {I_{{i{\text{C}}}} (2)} & {\begin{array}{*{20}c} \cdots & {I_{{i{\text{C}}}} (M)} \\ \end{array} } \\ \end{array} ]^{{\text{T}}} \\ & I_{i} = [\begin{array}{*{20}c} {I_{{i\text{A}}} } & {I_{{i{\text{B}}}} } & {\begin{array}{*{20}c} {I_{{i{\text{C}}}} } \\ \end{array} } \\ \end{array} ]^{{\text{T}}} \\ \end{aligned} \right.$$

where *i* = 1, 2, 3, …, *N*; *k* = 1, 2, 3, …, *M*. *I*_*i*A_(*k*), *I*_iB_(*k*), *I*_*i*C_(*k*) are the current amplitudes of the *k*th sampling point of the node *I *before the fault occurs, and *M* is the total number of data obtained by discretization.

After a length of time, the current *I*_*i*F_ of each node is derived using discretization:

31 $$\left\{ \begin{aligned} & I_{{i\text{FA}}} = [\begin{array}{*{20}c} {I_{{i\text{FA}}} (1)} & {I_{{i\text{FA}}} (2)} & \cdots & {I_{{i\text{FA}}} (M)} \\ \end{array} ]^{{\text{T}}} \hfill \\ & I_{{i\text{FB}}} = [\begin{array}{*{20}c} {I_{{i\text{FB}}} (1)} & {I_{{i\text{FB}}} (2)} & \cdots & {I_{{i\text{FB}}} (M)} \\ \end{array} ]^{{\text{T}}} \hfill \\ & I_{{i\text{FC}}} = [\begin{array}{*{20}c} {I_{{i\text{FC}}} (1)} & {I_{{i\text{FC}}} (2)} & \cdots & {I_{{i\text{FC}}} (M)} \\ \end{array} ]^{{\text{T}}} \hfill \\ & I_{{i{\text{F}}}} = [\begin{array}{*{20}c} {I_{{i{\text{FA}}}} } & {I_{{i{\text{FB}}}} } & {\begin{array}{*{20}c} {I_{{i{\text{FC}}}} } \\ \end{array} } \\ \end{array} ]^{{\text{T}}} \hfill \\ \end{aligned} \right.$$

where *i* = 1, 2, 3, …, *N*; *k* = 1, 2, 3, …, *M*. *I *_*i*FA_(*k*), *I*_*i*FB_(*k*), *I*_*i*FC_(k) are the current amplitudes of the *k*th sampling point of the node *i* after the fault.

To realize fault region identification under limited PMU, the SNRC is defined to measure the difference between the upstream and downstream currents of the fault point. The expression of SNRC is as follows:

32 $$K = \frac{{||I_{i} ||_{2} }}{{||I_{{i{\text{F}}}} ||_{2} }}$$

where, ||*I*_*i*_||_2_ represents the 2-norm of the current before the fault of node *i*, and ||*I*_*i*F_||_2_ represents the 2-norm of the current after the fault of node *i*. Then the SNRC variation Δ*K*_*m*_ of the region *m* (the first node and end node of region *m* are nodes *i* and *j*, respectively) is:

33 $$\Delta K_{m} = |K_{i} - K_{j} |$$

where *m* = 1, 2, 3, …, *J*; *i*, *j* = 1, 2, 3, …, *N*, and *i* ≠ *j*.

When the line is in normal operation, the 2-norm of the three-phase current calculated for the entire line for any period is not significantly different. Hence, the Δ*K* of each region is close to 0. When the line faults, the phase current is greater than the threshold *I*_set_, and the Δ*K* of each region increases. Moreover, the Δ*K* of the fault region is significantly greater than the Δ*K* of the normal region. As a result, the fault region may be identified by comparing the variations in Δ*K* of each location.

#### Current phase angle difference

Because there is no current flowing downstream of the fault point when a fault occurs in the downstream region without DG integration, the fault region may still be recognized using the SNRC. When a fault occurs in the downstream region with DG access, the DG injects current into the fault point. Current flows downstream of the fault point. Relying exclusively on the SNRC may lead to errors. Equations (26)–(28) shows that the CPD of the normal region is 0. However, the CPD of the fault region will be much more significant than that in the normal region. To identify the fault region, this study combines the CPD stated in Eq. (34) with the SNRC.

34 $$\left\{ \begin{aligned} & \Delta \varphi_{M} = \varphi_{M{\text{F}}} - \varphi_{M{\text{P}}} \hfill \\ & \Delta \varphi_{N} = \varphi_{N{\text{F}}} - \varphi_{N{\text{P}}} \hfill \\ & \Delta \varphi_{MN} = |\Delta \varphi_{M} - \Delta \varphi_{N} | \hfill \\ \end{aligned} \right.$$

where *φ*_MF_ and *φ*_*N*F_ are the current phases after node M and N fault, respectively.* φ*_*M*_ and *φ*_*N*P_ are the current phases before node M and N fault, respectively. Δ*φ*_*M*_ is the current phase variation of node M. Δ*φ*_*N*_ is the current phase variation of node N. Δ*φ*_*MN*_ is the CPD of the region (node M and node N are the first node and end node of the region, respectively.) If the region MN is normal, then its Δ*φ*_*MN*_ approaches 0. Inversely, its Δ*φ*_*MN*_ will be the maximum one.

### Fault region identification start criterion

In this paper, PMUs are utilized to monitor the phase current in real-time. Meanwhile, the exists of faults is determined based on whether the phase current exceeds the limit. The particular requirement is as follows:

35 $$I \ge I_{{\text{set}}}$$

where *I*_set_ is the fault protection threshold. The neutral point ungrounded system threshold *I*_set-U_ and the neutral point through the arc suppression coil grounding system threshold *I*_set-A_ respectively, meet:

36 $$\left\{ \begin{aligned} & I_{{\text{set - U}}} = 0.5K_{{\text{rel}}} (I_{C\Sigma } - I_{L} ) \hfill \\ & I_{{\text{set - A}}} = 0.5WK_{{\text{rel}}} I_{C\Sigma } \hfill \\ \end{aligned} \right.$$

where, *K*_rel_ is the reliability coefficient, generally 0.8; *I*_CΣ_ is the total ground capacitive current; *I*_L_ is the maximum line-to-ground capacitance current; and *W* is the over-compensation degree.

When the above starting criterion is satisfied at time *t*, PMU will take the point at time *t* as the fault starting point and record the phase current data before the fault starting point and the phase current data of a cycle after the fault starting point. According to the data, the PMUs at both ends of the region use Eqs. (32)–(34) to identify the fault region.

To eliminate the impact of other interference signals on the beginning fault region identification, the PMU is utilized to capture phase current data one cycle after the fault starting point for verification after finding the fault starting point. If Eq. (37) is fulfilled, the identification and the next step are determined.

37 $$\left\{ \begin{aligned}{}& I_{km} \ge KI_{n} \hfill \\ & I_{km} = \sqrt {\frac{1}{N}\sum\limits_{k = 1}^{N} {i^{2}_{n} (k)} } \hfill \\ \end{aligned} \right.$$

where, *I*_km_ is the practical phase current value in one cycle, and *K* is the reliability coefficient of phase current fault, generally *K* = 2–3. *I*_n_ is the reasonable value of phase current when the line is normal, and *i*_n_(*k*) is the instantaneous value of phase current at the *k*th identification point after the fault.

### PMU configuration and region division strategies

**Figure 5:**
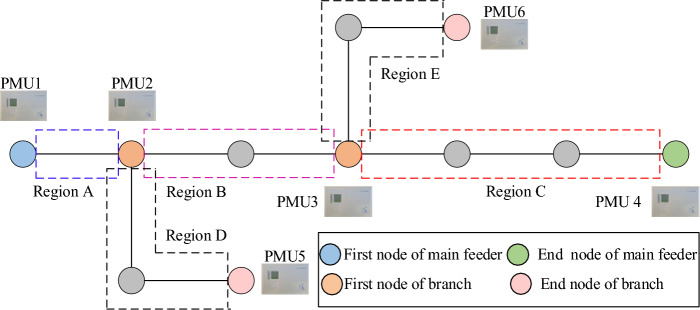
Diagram of PMU configuration and region division.

The PMU configuration and region division strategies are proposed in this paper, as shown in Fig. 5. Specifically, the PMU configuration strategy is as follows:

1. The first node of main feeder and the end node of main feeder in ADN are arranged with PMU, respectively;
2. The first node of branch and the end node of branch are arranged with PMU, respectively.

In addition, the region division strategy is:

1. The first node of main feeder and the first node of the nearest brunch in distribution network is the regional boundaries;
2. The first node of branch and the end node of the branch is the regional boundary;
3. The end node of main feeder and the first node of the nearest branch is the regional boundaries.

## Fault region identification method steps

**Figure 6:**
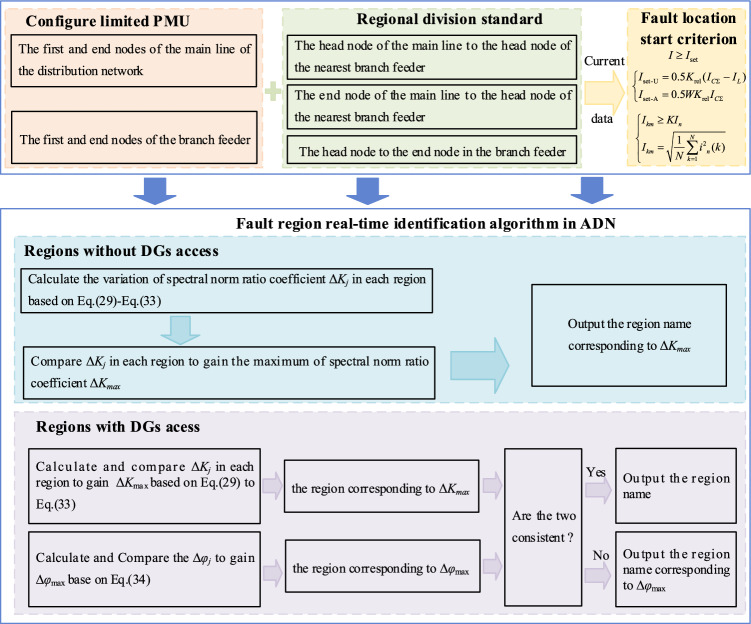
The framework of the proposed method.

**Figure 7:**
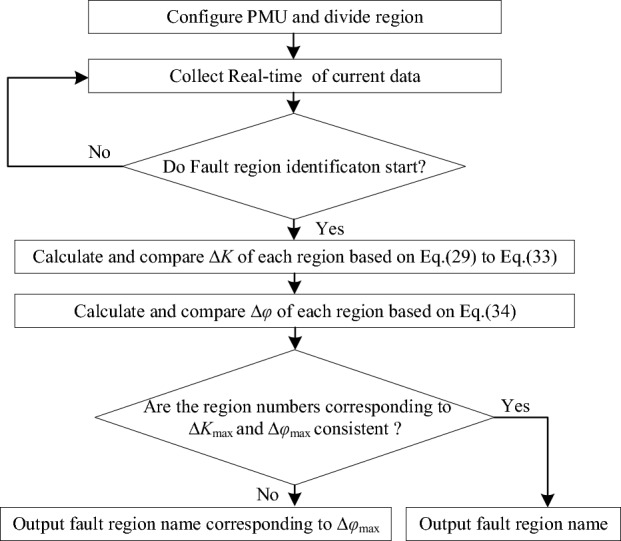
The flowchart of the proposed method.

The framework and flowchart of the proposed method shown in Figs. 6-7. The implementation steps are as follows:

Step 1: arrange the PMUs according to the proposed method and divide the region.
Step 2: use the three-phase current data collected by PMUs. Judge the fault according to Eqs. (35)–(37).
Step 3: calculate and compare Δ*K*_*j*_ of each region based on Eqs. (29)–(33) to gain the region corresponding to the Δ*K*_max_.
Step 4: calculate and compare Δ*φ*_j_ of each region based on Eq. (34).
Step 5: if the region corresponding to Δ*K*_max_ is the same as that corresponding to ∆*φ*_max_, output the region name. Adversely, output the region corresponding to ∆*φ*_max_.

## Simulation results

**Figure 8:**
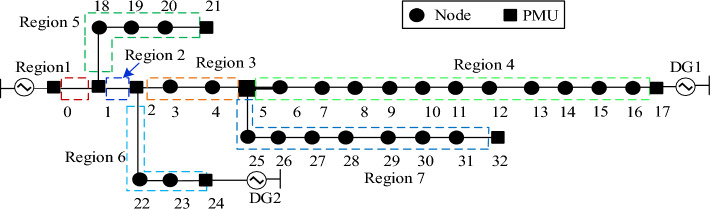
IEEE 33-node distribution network.

The IEEE 33-node system is built in PSCAD/EMTDC, using a simulation sampling frequency of 4 kHz. The parameters of the system are shown in the Supplementary Information. The capacities of DG1 and DG2 are 0.544 MVA and 1.4 MVA, respectively. MatlabR2016b is utilized for data analysis and computation to verify the effectiveness of the proposed fault region identification method. According to the method of PMU layout and region division, the nodes and region division of PMU in an IEEE 33-node distribution network are shown in Fig. 8.

**Figure 9:**
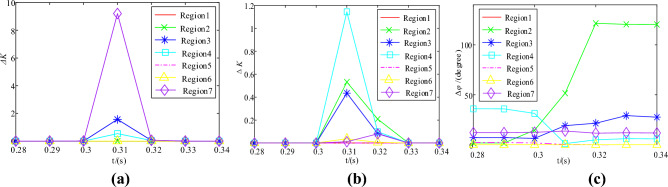
Δ*K* and Δ*φ* in each region in case of fault in Region 7 and Region 5 respectively. (**a**) Δ*K* for each region in case of fault in Region 7; (**b**) Δ*K* for each region in case of fault in Region 5; (**c**) Δ*φ* for each region in case of fault in Region 5.

Firstly, set an A-phase grounding fault in Region 7 and combine it with the phase current data collected by PMU. The curve of Δ*K* variation with time in each region is obtained, as depicted in Fig. 9(a). Secondly, set up a phase A grounding fault in Region 2 and use the data collected by PMU. The Δ*K* variation and CPD with time in each region is obtained, as shown in Fig. 9(b)-(c).

Figure 9(a) shows that the Δ*K*_max_ occurs when a fault occurs in a region without DG access downstream. Additionally, Fig. 9(b)-(c) show that when a fault occurs in a region with DG access downstream, its Δ*K* is second only to Δ*K*_max_, but the CPD is at its maximum. To accurately identify the fault region, the SNRC and CPD of the region can be combined.

**Figure 10:**
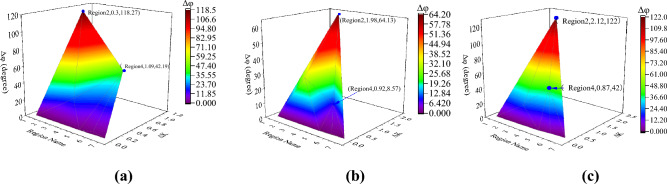
Identification results under different fault types in Region 2. (**a**) Single-phase fault; (**b**) Two-phase fault; (**c**) Three-phase fault.

**Figure 11:**
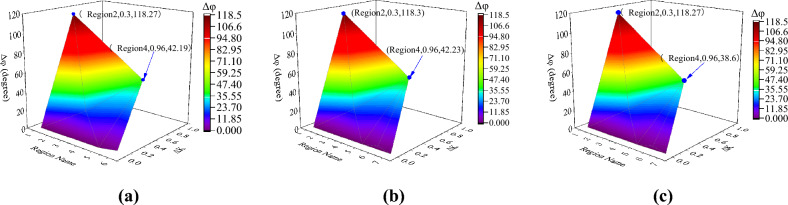
Identification results of Region 2 fault under different neutral grounding modes. (**a**) Neutral point ungrounded mode; (**b**) Neutral point through low resistance grounding mode; (**c**) Neutral point grounding through arc suppression coil.

To verify the reliability and robustness of the method, the fault type, neutral point grounding mode, and fault transition resistance of the line are changed, respectively. The results are illustrated in Figs. 10-11 and Tables 1-2. From the results in Fig. 10, the SNRC variation and CPD of Region 2 reach their maximum when two-phase faults and three-phase faults occur in Region 2. This is because the short-circuit current delivered by DG1 and DG2 is slight when a two-phase or three-phase fault occurs.

**Table 1:** Identification results of Region 5 fault under different transition resistance.

Transition resistance	Δ*K/*Δ*φ*	Region 1	Region 2	Region 3	Region 4	Region 5	Region 6	Region 7	Identification results
50Ω	Δ*K*	0	0.0016	0	0.0085	5.7765	0.0003	0.0013	Region 5
	Δ*φ*	0°	1.46°	1.46°	3.75°	21.37°	0.22°	0.01°	
700Ω	Δ*K*	0	0.002	0.0006	0.001	0.3790	0	0.0001	Region 5
	Δ*φ*	0°	0.66°	0.33°	0.51°	8.75°	0.02°	0.01°	
3000Ω	Δ*K*	0	0.0005	0.0002	0.0002	0.0780	0	0	Region 5
	Δ*φ*	0°	0.16°	0.01°	0.05°	0.21°	0°	0.01°

**Table 2:** Identification results of Region 2 fault under different transition resistance.

Transition resistance	Δ*K/*Δ*φ*	Region 1	Region 2	Region 3	Region 4	Region 5	Region 6	Region 7	Identification results
50Ω	Δ*K*	0	0.0054	0.0002	0.0111	0	0.0004	0.0014	Region 2
	Δ*φ*	0.01°	8.84°	1.68°	4.09°	0.08°	0.16°	0.01°	
700Ω	Δ*K*	0	0.0019	0.0007	0.0011	0	0	0.0001	Region 2
	Δ*φ*	0.02°	0.66°	0.16°	0.32°	0.02°	0.12°	0.02°	
3000Ω	Δ*K*	0	0.0005	0.0002	0.0003	0	0	0	Region 2
	Δ*φ*	0°	0.15°	0.08°	0.07°	0.03°	0.02°	0.02°	

Figure 11 shows the identification results under various neutral point grounding modes, indicating that the grounding mode of the neutral point has little effect on the proposed method and that the fault region can still be accurately identified under various neutral grounding modes.

The results and analyses in Tables 1-2 reveal that the method has a decent identification impact in any location. The smaller the Δ*K* and Δ*φ* of each region, the more excellent the transition resistance. Furthermore, the SNRC variation of the fault region will reach their maximum values. This is because the short-circuit current of the distributed power supply is reduced when the transition resistance increases. As a result, in the case of a high impedance fault, the method proposed can still identify the fault region.

Table 3 demonstrates the identification result with the PMU installed in node 2 takes 10 ms longer to gather data than other PMUs. It can be obtained that when a single-phase ground fault occurs in Region 7, the Δ*K* and Δ*φ* of Region 7 are greater than those in other regions. While a single-phase ground fault occurs in Region 2, the Δ*φ* of Region 2 reaches the maximum. And the Δ*K* of Region 2 is the second largest compared with other regions. This is because the estimated current spectrum norm measures the current's variation in a data window, whereas the current phase utilizes the steady-state value. As a result, the method suggested in this research has low communication needs between PMUs.

**Table 3:** Fault region identification results of node 2 with a PMU delay of 10 ms.

Fault region	Δ*K/*Δ*φ*	Region 1	Region 2	Region 3	Region 4	Region 5	Region 6	Region 7	Identification results
Region 7	Δ*K*	0	0.0139	1.6856	0.5771	0	0.0013	8.9431	Region 7
	Δ*φ*	0°	0.25°	47.83°	78.86°	0°	0.29°	83.77°	
Region 2	Δ*K*	0	1.0991	1.6581	0.9554	0.0003	0.0287	0.0127	Region 2
	Δ*φ*	0.01°	118.23°	20.82°	42.19°	0.51°	2.44°	0.35°	

**Figure 12:**
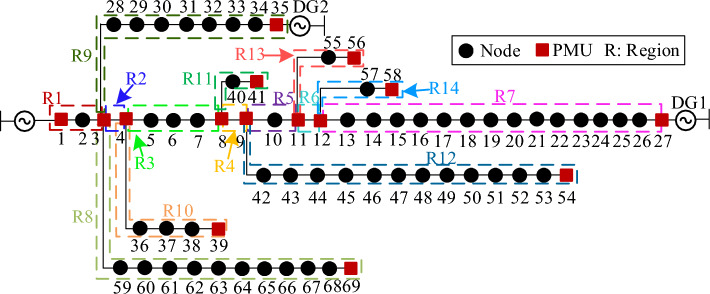
IEEE 69-node distribution network.

In order to verify the effectiveness of the proposed method in large-scale distribution networks, this paper builds an IEEE 69-node distribution network system as shown in Fig. 12. The parameters of the system are shown in the Supplementary Information. The capacities of DG1 and DG2 are 0.544 MVA and 1.4 MVA, respectively. Set an A-phase grounded fault in R7(node 12-node 27) and R8(node 3-node 69), respectively. Collect the current data of each cycle before and after the fault to calculate the Δ*K* of each region. The result is shown in Table 4. It can be obtained that when a fault occurs in the region downstream of the fault point accessing the DG (e.g., R7), the Δ*φ* of the fault region are significantly larger than those of the other regions. And the Δ*K* will reach the maximum or the second largest. When a fault occurs in the region downstream of the fault point without access to the DG (e.g., R8), the Δ*K* and Δ*φ* in the fault region are both significantly larger than those in normal regions. Therefore, the method proposed in this paper is still able to effectively and accurately identify fault region under large-scale distribution networks.

**Table 4:** Identification results of R7 and R8 fault respectively.

Region name	Fault region				Region name	Fault region			
	R7		R8			R7		R8	
	Δ*K*	Δ*φ*	Δ*K*	Δ*φ*		Δ*K*	Δ*φ*	Δ*K*	Δ*φ*
R1	0	0°	0.0016	0°	R8	0	0.018°	0.2783	12.959°
R2	0.0003	0.214°	0.0028	1.757°	R9	0.001	0.558°	0.0131	2.43°
R3	0.0001	0.314°	0.0017	0.008°	R10	0.0018	0.016°	0.0135	0.044°
R4	0.0021	0.841°	0.0048	0.108°	R11	0.0001	0.001°	0.0006	0.003°
R5	0.0032	4.162°	0.0021	0.388°	R12	0.002	0.013°	0.0251	0.001°
R6	0.0467	44.58°	0.0007	2.82°	R13	0.0822	0°	0.0217	0°
R7	0.1217	53.724°	0.0032	2.001°	R14	0	0.001°	0	0°

To further verify the superiority of the methods proposed, this paper compared it with other four methods. The four methods use the characteristics of the upstream and downstream currents at the fault point to realize fault identification. Reference^20^ realizes fault region identification with the help of zero sequence conductance. Although it is independent of neutral grounding method, transition resistance and communication desynchronization, it can only identify single-phase faults and can only be applied to single-supply systems. Literature^21^, compared to literature^20^, is independent of fault type, but it requires communication synchronization. The methods proposed in literatures^22,23^ are applicable to DG-integrated distribution networks, but do not consider the effects of neutral grounding method, transition resistance and communication desynchronization. The specific method comparison results are shown in Table 5. Therefore, method proposed in this paper is more comprehensively considered and superior to other methods.

**Table 5:** The main characteristics of other methods and the method proposed in this paper.

Reference	20	21	22	23	This paper
Fault type	SPF	ALL	ALL	SPF&TPF	ALL
Neutral point grounding method	ALL-N	ALL-N	×	×	ALL-N
High impedance fault	√	√	×	×	√
Source type	SS	SS	MS	MS	MS
Communications asynchrony	√	×	×	×	√

Symbol meanings in this table:

ALL/SPF/TPF: all faults/single-phase fault/two-phase fault.

ALL-N: all neutral point grounding method.

SS/MS: single source/multiple sources.

√/ × : this aspect is/no considered.

## Experiment results

**Figure 13:**
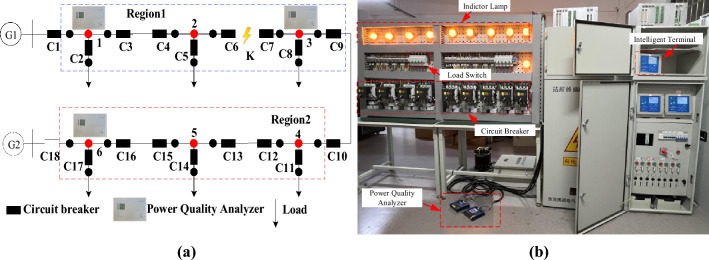
Test platform (**a**) The topology of the test platform; (**b**) The actual test platform.

To verify the efficacy and dependability of the proposed method, a real 6-node test platform is used to validate the method. Figure 13 depicts the test platform structure, PMU configuration, and region division. The power quality analyzer replaces the PMU in the actual test, and three power quality analyzers acquire the platform’s current data before and after the short-circuit defect at a 4 kHz acquisition frequency. Simultaneously, a sampling time of 10 ms is set between the three power quality analyzers mentioned above to examine the proposed method's performance change when the PMU clock is not synced.

**Figure 14:**
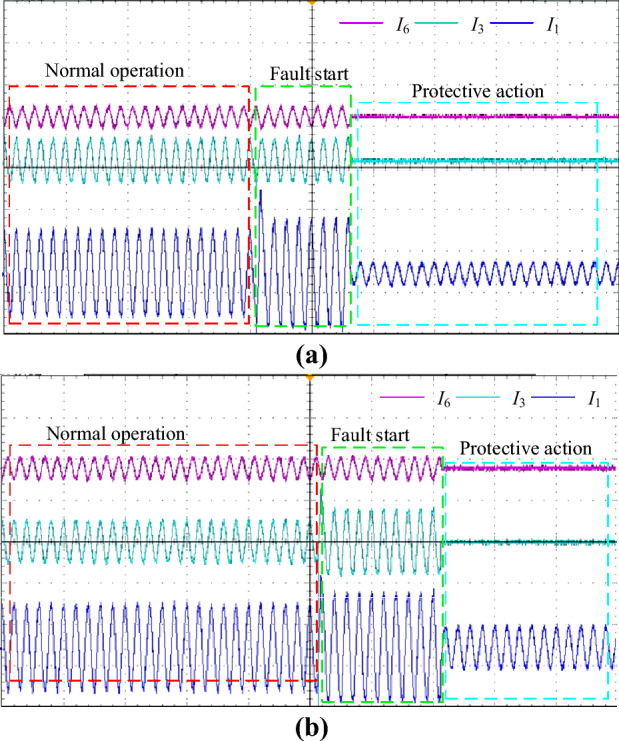
Measured current waveform of each node (**a**) Region 1 faults; (**b**) Region 2 faults.

In the actual experiment, the fault is set in Region 1 and Region 2, respectively. Figure 14 depicts the measured waveforms of the currents at both ends of the region when Region 1 and Region 2 fault respectively. It can be found that when Region 1 faults, the current at node 1 increases and the currents at node 3 and node 6 remain essentially unchanged. Whereas, when Region 2 faults, the currents at both node 1 and node 2 increase and the current at node 6 remains essentially unchanged. The fault region identification results obtained by the proposed method are shown in Table 6. It can be obtained that the Δ*K* and Δ*φ* of the faulty region are significantly larger than those of the normal region when either Region 1 or Region 2 fault. Thus, the method proposed in this paper can still accurately identify the fault region when the communication is not synchronized, proving its practical potential.

**Table 6:** Fault region identification results of Region 1 and Region 2 fault respectively.

Fault region	Δ*K/*Δ*φ*	Region1	Region2	Identification results
Region 1	Δ*K*	0.2799	0.0242	Region 1
	Δ*φ*	132.7°	67.1°	
Region 2	Δ*K*	0.3109	0.5960	Region 2
	Δ*φ*	68.3°	159.3°	

## Conclusion

The paper analyzes the fault characteristics of the ADN in detail. A fault region identification approach based on the SNRC and CPD is proposed for the ADN. It only needs to extract the current amplitude and phase data of any period before and after the fault occurs, avoiding the impact of time synchronization on fault region identification. Case studies of faults under different conditions are used to evaluate the performance of the proposed method. The results demonstrate that the method is less affected by fault type, neutral grounding mode, fault position, transition resistance, and communication. Moreover, the actual test platform shows that the proposed method can effectively identify the fault region and has practical significance.

With the further development of synchronous measuring technology, PMU will become more commonly employed in distribution networks. Understanding how to use restricted PMU to accomplish accurate fault location in the ADN is critical.

## Supplementary Information

Supplementary Tables.

## Data Availability

The datasets used and/or analyzed during the current study available from the corresponding author on reasonable request.
