# Supplementary material for: Current spectral norm and phase variation based fault region identification for active distribution network
Source: Sci Rep. 2024 Jun 2;14:12640. doi: 10.1038/s41598-024-62859-6 (PMC11637091; doi:10.1038/s41598-024-62859-6)
Supplement: Supplementary file 1 — Supplementary Tables. [file 41598_2024_62859_MOESM1_ESM.docx]

**Supplementary Material**

(1) Parameters of 33-node distribution network system

Tab.1 Parameters of IEEE 33-node distribution network system

| Node *i* | Node *j* | Branch impedance/Ω | Node *j* load/(kVA) | Node *i* | Node *j* | Branch impedance/Ω | Node *j* load/(kVA) |
| --- | --- | --- | --- | --- | --- | --- | --- |
| 0 | 1 | 0.0922+j0.047 | 100+j60 | 16 | 17 | 0.372+j0.574 | 90+j40 |
| 1 | 2 | 0.493+j0.2511 | 90+j40 | 1 | 18 | 0.164+j0.1565 | 90+j40 |
| 2 | 3 | 0.366+j0.1864 | 120+j80 | 18 | 19 | 1.5042+j1.3544 | 90+j40 |
| 3 | 4 | 0.3811+j0.1941 | 60+j30 | 19 | 20 | 0.4095+j0.4784 | 90+j40 |
| 4 | 5 | 0.8190+j0.707 | 60+j20 | 20 | 21 | 0.7089+j0.9373 | 90+j40 |
| 5 | 6 | 0.1872+j0.6188 | 200+j100 | 2 | 22 | 0.4512+j0.3083 | 90+j50 |
| 6 | 7 | 0.7114+j0.2351 | 200+j100 | 22 | 23 | 0.898+j0.7091 | 420+j200 |
| 7 | 8 | 1.03+j0.74 | 60+j20 | 23 | 24 | 0.8960+j0.7011 | 420+j200 |
| 8 | 9 | 1.044+j0.74 | 60+j20 | 5 | 25 | 0.203+j0.1034 | 60+j25 |
| 9 | 10 | 0.1966+j0.065 | 45+j30 | 25 | 26 | 0.2842+j0.1447 | 60+j25 |
| 10 | 11 | 0.3744+j0.1238 | 60+j35 | 26 | 27 | 1.059+j0.9337 | 60+j20 |
| 11 | 12 | 1.468+j1.155 | 60+j35 | 27 | 28 | 0.8042+j0.7006 | 120+j70 |
| 12 | 13 | 0.5416+j0.7129 | 120+j80 | 28 | 29 | 0.5075+j0.2585 | 200+j600 |
| 13 | 14 | 0.591+j0.526 | 60+j10 | 29 | 30 | 0.9744+j0.9630 | 150+j70 |
| 14 | 15 | 0.7463+j0.545 | 60+j20 | 30 | 31 | 0.3105+j0.3619 | 210+j100 |
| 15 | 16 | 1.2890+j1.721 | 60+j20 | 31 | 32 | 0.341+j0.5362 | 60+j40 |

(2) Parameters of 69-node distribution network system

Tab.2 Parameters of IEEE 69-node distribution network system

| Node *i* | Node *j* | Branch impedance/Ω | Node *j* load/(kVA) | Node *i* | Node *j* | Branch impedance/Ω | Node *j* load/(kVA) |
| --- | --- | --- | --- | --- | --- | --- | --- |
| 1 | 2 | 0.005+j0.0012 | 0 | 34 | 35 | 1.474+j0.4873 | 6+j4 |
| 2 | 3 | 0.005+j0.0012 | 0 | 3 | 59 | 0.0044+j0.0108 | 26+j18.55 |
| 3 | 4 | 0.0015+j0.0036 | 0 | 59 | 60 | 0.064+j0.1565 | 26+j18.55 |
| 4 | 5 | 0.0251+j0.0294 | 0 | 60 | 61 | 0.1053+j0.123 | 0 |
| 5 | 6 | 0.366+j0.1864 | 2.6+j2.2 | 61 | 62 | 0.0304+j0.0355 | 24+j17 |
| 6 | 7 | 0.3811+j0.1941 | 40.4+j30 | 62 | 63 | 0.0018+j0.0021 | 24+j17 |
| 7 | 8 | 0.0922+j0.047 | 75+j54 | 63 | 64 | 0.7283+j0.8509 | 1.2+j1 |
| 8 | 9 | 0.0493+j0.0251 | 30+j22 | 64 | 65 | 0.31+j0.3623 | 0 |
| 9 | 10 | 0.819+j0.2707 | 28+j19 | 65 | 66 | 0.041+j0.0478 | 6+j4.3 |
| 10 | 11 | 0.1872+j0.0691 | 145+j104 | 66 | 67 | 0.0092+j0.0116 | 0 |
| 11 | 12 | 0.7114+j0.2351 | 145+j104 | 67 | 68 | 0.1089+j0.1373 | 39.22+j26.3 |
| 12 | 13 | 1.03+j0.34 | 8+j5.5 | 68 | 69 | 0.0009+j0.0012 | 39.22+j26.3 |
| 13 | 14 | 1.044+j0.345 | 8+j5.5 | 4 | 36 | 0.0034+j0.0084 | 0 |
| 14 | 15 | 0.1966+j0.065 | 0 | 36 | 37 | 0.0851+j0.2083 | 79+j56.4 |
| 15 | 16 | 0.1966+j0.065 | 45.5+j30 | 37 | 38 | 0.2898+j0.7091 | 384.7+j274.5 |
| 16 | 17 | 0.3744+j0.1238 | 60+j35 | 38 | 39 | 0.0822+j0.2011 | 384.7+j274.5 |
| 17 | 18 | 0.0047+j0.0016 | 60+j35 | 8 | 40 | 0.0928+j0.0473 | 40.5+j28.3 |
| 18 | 19 | 0.3276+j0.1083 | 0 | 40 | 41 | 0.3319+j0.1114 | 3.6+j2.7 |
| 19 | 20 | 0.2106+j0.0696 | 1+j0.6 | 9 | 42 | 0.174+j0.0886 | 4.35+j3.5 |
| 20 | 21 | 0.3416+j0.1129 | 114+j81 | 42 | 43 | 0.203+j0.1034 | 26.4+j19 |
| 21 | 22 | 0.014+j0.0046 | 5.3+j3.5 | 43 | 44 | 0.2842+j0.1447 | 24+j17.2 |
| 22 | 23 | 0.1591+j0.0526 | 0 | 44 | 45 | 0.2813+j0.1433 | 0 |
| 23 | 24 | 0.3463+j0.1145 | 28+j20 | 45 | 46 | 1.59+j0.5337 | 0 |
| 24 | 25 | 0.7488+j0.2457 | 0 | 46 | 47 | 0.7837+j0.263 | 0 |
| 25 | 26 | 0.3089+j0.1021 | 14+j10 | 47 | 48 | 0.3042+j0.1006 | 100+j72 |
| 26 | 27 | 0.1732+j0.0572 | 14+j10 | 48 | 49 | 0.3861+j0.1172 | 0 |
| 3 | 28 | 0.0044+j0.0108 | 26+j18.6 | 49 | 50 | 0.5075+j0.2585 | 1244+j888 |
| 28 | 29 | 0.064+j0.1565 | 26+j18.6 | 50 | 51 | 0.0974+j0.0496 | 32+j23 |
| 29 | 30 | 0.3978+j0.1315 | 0 | 51 | 52 | 0.1450+j0.0738 | 0 |
| 31 | 32 | 0.351+j0.116 | 0 | 52 | 53 | 0.7105+j0.3619 | 227+j162 |
| 32 | 33 | 0.839+j0.2816 | 0 | 53 | 54 | 1.041+j0.5302 | 59+j42 |
| 33 | 34 | 1.708+j0.5646 | 14+j10 | 11 | 55 | 0.2012+j0.0611 | 18+j13 |
| 55 | 56 | 0.0047+j0.0016 | 18+j13 | 12 | 57 | 0.7394+j0.2444 | 28+j20 |
| 57 | 58 | 0.0047+j0.0016 | 28+j20 |  |  |  |  |
